# A Comparison of Clinical Diagnostic Classification Criteria Used in Longitudinal Cohort Studies of the Alzheimer’s Disease Continuum: A Systematic Review

**DOI:** 10.1007/s11065-025-09663-9

**Published:** 2025-05-09

**Authors:** Juan Manuel Villalpando, Bernard-Simon Leclerc, Minh Tri Le, Carol Hudon, Aline Bolduc, Marie-Jeanne Kergoat, Consortium for the Early Identification of Alzheimer’s Disease-Quebec (CIMA-Q)

**Affiliations:** 1 Centre de recherche de l’Institut universitaire de gériatrie de Montréal (IUGM), Centre intégré universitaire de santé et de services Sociaux (CIUSSS) du Centre-Sud-de-l’Île-de-Montréal, Montréal, Canada; 2 Faculté de Médecine, Université de Montréal, Montréal, Canada; 3 École de Psychologie, Université Laval, Québec, Canada; 4 Centre de Recherche CERVO, Québec, Canada; 5 Département de Médecine Sociale et Préventive, École de Santé Publique, Université de Montréal, Montréal, Canada

**Keywords:** Alzheimer’s disease, Cognitive tests, Diagnosis, Mild cognitive impairment, Cohort studies, Systematic review

## Abstract

Alzheimer’s is a progressive disease, with a long preclinical phase of many decades. Accurate classification within longitudinal cohort studies is crucial for understanding disease progression and for the comparability and collaboration across studies. The main objective of this systematic review was to identify and compare the diagnostic criteria used in prospective population study cohorts centering on the Alzheimer’s disease clinical continuum in older adults. A review was performed of cohort studies started in the year 2000 or later, with a follow-up duration of at least 3 years among people aged between 50 and 85 years old living in the community. Original studies were searched in MEDLINE, Embase, Cochrane, PsycINFO, and Web of Science. Two independent reviewers agreed on the final selection of 28 studies covering 25 cohorts. One study was identified by three independent judges as having methodological limitations due to inadequate reporting as per the modified NIH quality assessment tool. Data was extracted from each included study using a standardized extraction form. In general, the studies followed fewer than 1500 participants. The results showed convergence in the choice of diagnostic classification criteria among the 25 cohorts studied especially for the later stages of AD, while criteria for the earliest stages showed greater variability. Only five cohorts studied were concerned with the follow-up of the full spectrum of the disease. Our study may help to put in place a unified set of clinical diagnostic criteria across the continuum of Alzheimer’s disease, rather than criteria developed specifically for a given study.

**Supplementary Information:**

The online version contains supplementary material available at 10.1007/s11065-025-09663-9.

## Introduction

Alzheimer’s disease (AD) is a major health issue affecting the older adult population worldwide. It is estimated that 32 million individuals around the world have AD dementia, and that 22% of those over the age of 50 are currently living with preclinical or prodromal AD (Gustavsson et al., 2023). Dementia is a leading cause of death and disability in older adults. The impact of AD extends beyond the diagnosed individuals. This is reflected in increased healthcare demands and a reduction in quality of life, as well as having a large-scale societal and economic impact (Wimo et al., 2023).

AD is a progressive disease, with a long preclinical phase. Its first cognitive changes can be seen as far back as 20 years before a clinical diagnosis of dementia is made (Amieva et al., 2014; Jia et al., 2024). While the progression of AD is a continuous process, there are different clinical stages in the Alzheimer continuum, going from the earliest prodromal manifestations to full-blown dementia. These stages can be considered as milestones in the disease, going from subjective cognitive decline (SCD, subjective cognitive complaints without objective evidence of decline on cognitive testing and with no impact on activities of daily living), mild cognitive impairment (MCI, broadly defined as the presence of objective cognitive changes without significant impact on activities of daily living) to dementia (objective cognitive impairment with associated progressive decline in activities of daily living). The study of the early period of this clinical disease is warranted, since early-stage diagnosis and addressing modifiable risk factors could have a major effect on the prevalence of this disorder and its consequences (Livingston et al., 2020).

AD has been traditionally diagnosed by the use of internationally accepted criteria, which include clinical, cognitive, and functional features. Still, the limitations of a clinical diagnosis of AD are well known. For example, a study by Serrano-Pozo (Serrano-Pozo et al., 2014) demonstrated that up to 14% of individuals clinically diagnosed with AD showed no or sparse neuritic plaques on autopsy, and a study by Ossenkoppele (Ossenkoppele et al., 2013) showed that only 61% of patients from a memory clinic with a clinical diagnosis of Alzheimer’s disease had biological evidence of the disease, as demonstrated by a positive amyloid PET scan. The issue of misdiagnosis is compounded by the fact that there are more than one set of internationally accepted criteria for AD, which are similar but not superposable, and there is a high degree of diagnostic discordance between them. This is also seen when comparing older and newer versions of the same criteria. Bieger (Bieger et al., 2024) compared AD diagnosis in the ADNI cohort according to NIA-AA 2011 (McKhann et al., 2011), NIA-AA 2018 (Jack et al., 2018), IWG- 2 2016 (Dubois et al., 2016), and IWG- 3 2021 criteria (Dubois et al., 2021). They showed a discordant diagnosis in 42.8% of individuals across all guidelines, mostly in those with only a single positive biomarker (either βA or tau positivity). Therefore, the choice of clinical criteria used by a cohort study, as well as variations in how these criteria are applied across studies, such as differences in the selection of cognitive testing batteries, can limit or complicate the comparison and generalization of findings between cohorts.

The current shift towards a biological, i.e., biomarker based (Alzheimer’s Association (Jack et al., 2024)) or clinical-biological (International Working Group (Dubois et al., 2024)) definition of Alzheimer’s disease is a fundamental conceptual change in the diagnosis of AD, one which will help correct this significant risk for misdiagnosis going forward. However, most of the ongoing or recently completed cohort studies still rely on clinical criteria or methodologies, which raise challenges when comparing data across research cohorts.

In response to these challenges, our goal was to carry out a systematic review to identify the convergence, divergence, or heterogeneity of the different clinical diagnostic criteria used in longitudinal cohort studies which focus on the evolution of cognitive changes on the Alzheimer’s disease continuum (i.e., ranging from cognitively normal individuals to individuals with dementia). We also sought to compare our own ongoing cohort, the Consortium for the Early Identification of Alzheimer’s Disease (CIMAQ, according to its French acronym) (Belleville et al., 2019) with other recent cohort studies focusing on similar populations worldwide.

## Methods

This systematic review adheres to the PRISMA (Preferred Reporting Items for Systematic reviews and Meta-Analyses) 2020 statement (Page et al., 2021). A meta-analysis was not applicable in this study as it focused on a non-quantitative topic. The protocol of this review was not registered in any database.

### Inclusion and Exclusion Criteria

Individual studies were included according to the following criteria: (1) any prospective, descriptive, or analytical longitudinal cohort study examining the clinical progression to AD dementia; (2) carried out among people aged between 50 and 85 years old living in the community, as the focus of this study was sporadic AD; (3) started in the year 2000 or later, since the concept of mild cognitive impairment (MCI) was not well defined until the late 1990 s; (4) with a planned or actual follow-up duration of at least 3 years, since the focus of our review is on longitudinal cohorts; (5) published in peer-reviewed journals up until June 27 th, 2022, in either English or French.

Studies were excluded if: (1) there was no mention of the criteria used to diagnose the cognitive disorders in the AD continuum; (2) the diagnosis of AD was based on the result of a single neuropsychological test; use of the Clinical Dementia Rating (CDR) as a single instrument was permitted; (3) the diagnosis was made without a standardized protocol, which should ideally encompass a clinical assessment conducted by a physician or collaboratively by a physician and a neuropsychologist; (4) the study also followed other neurological or neurodevelopmental disorders (e.g., dementia associated with HIV, Parkinson’s disease, Down syndrome) outside the AD continuum; (5) the study was a sub-branch or sub-analysis of a parent cohort, in which case only the source cohort study was eligible for inclusion; (6) the cohort study was not prospectively designed for the assessment, classification, and monitoring of neurocognitive disorders; (7) the study combined participants from different cohorts; (8) the study was designed to address a specific question or intervention (e.g., the association between retinal thickness and cognitive impairment); (9) the study recruited only individuals with normal cognition and/or was not concerned with identifying or following individuals with subjective cognitive decline (SCD) or MCI, the intermediate stages of the clinical AD continuum; (10) the initial study population comprised less than 100 individuals since the focus of this review is on larger cohorts; and (11) the full article was not available.

### Information Sources and Search Strategy

The search strategy was reviewed, refined, and finalized with the help of a medical librarian experienced in database searches. It was based on the PICOS approach and was focused on: community dwelling individuals aged between 50 and 85 years old [Population]; tests and procedures used for the clinical diagnosis of cognitive disorders in AD progression [Intervention]; single or multiple groups composed of persons identified as being cognitively healthy, SCD, MCI, or AD dementia [Comparators]; aspects of the decision-making process for clinical diagnosis, i.e., what diagnostic criteria were used, how these were applied and how disagreements were resolved, classification categories of cognitive impairment in the clinical AD continuum, classification of cognitive status after initial assessment, and a minimum 3-year follow-up with at least two data collection points [Outcomes]; non-institutionalized people, living in the community (home or seniors’ residence) [Setting]. We employed a comprehensive search of titles, abstracts, and keywords in MEDLINE, Embase (OVID), Cochrane, PsycINFO, and Web of Science, covering the period from January 1, 2000, to June 27, 2022. The detailed search strategy is available in the Supplementary material (Appendix A). Additionally, we examined the bibliographies of all selected papers for further relevant studies. All 58 source articles associated with the studies comprised in the Cohort Studies of Memory in an International Consortium (COSMIC) registry were also reviewed (Cohort Studies of Memory in an International Consortium, 2023, https://cheba.unsw.edu.au/consortia/cosmic). This Consortium compiles data from various population-based longitudinal cohort studies focused on examining the determinants of cognitive aging, neurocognitive disorders, and dementia or cognitive decline.

### Study Selection Process

The screening and selection process for any study considered for inclusion in this review, according to the abovementioned search strategy, was carried out in two steps. Using the Covidence software (Melbourne, Australia), two independent reviewers (JMV and MTL) conducted an initial assessment of potential articles based on titles and abstracts. Discrepancies were resolved by consensus or, if necessary, with the help of a third reviewer (MJK). In the subsequent step, the same two main reviewers performed a full-text reading of the articles retained, determining inclusion or exclusion based on eligibility criteria. All articles meeting the eligibility criteria proceeded to the next phase for data extraction and analysis.

### Data Extraction Process

Full texts of selected papers were independently examined by the two main reviewers (JMV and MTL). Each one was assigned half of the chosen articles and they separately extracted nominal data from each study using a standardized template created in Covidence (Melbourne, Australia). This included information about the general cohort description, participant details, clinical diagnosis criteria, and evaluation protocols (see Supplementary Material, Table S1). If the information was not explicitly mentioned in the article, we contacted by e-mail the corresponding authors of these studies in order to obtain the official start date of each study, and the end date if the study was no longer in progress.

### Study Quality and Risk of Bias Assessment

The methodological quality of all selected studies was evaluated independently by two reviewers (JMV and MTL) using the National Institutes of Health (NIH) Quality Assessment Tool for Observational Cohort and Cross-Sectional Studies (National Heart, Lung and Blood Institute (NIH), 2021, https://www.nhlbi.nih.gov/health-topics/study-quality-assessment-tool (consulted 2022–11–17)), a validated instrument comprising 14 questions focusing on internal validity. The risk for potential selection, information, measurement, and confounding biases was estimated for every article. Each item received a response of “yes,” “no,” or an undifferentiated response if the information was not assessed, could not be determined, or was not reported in the article. Each reviewer compared the evaluation results of the other on a sample of randomly selected articles, and discrepancies were resolved through discussion and consensus. Responses from all assessments were ultimately checked for accuracy and completeness by an epidemiologist experienced in systematic reviews (BSL) who closely reviewed the articles. Three questions were added to evaluate specific items related to the aim of our review: (1) Was the recruitment method well described (were individuals referred from the community or from a family doctor or specialist)?; (2) Were the basic characteristics of the participants (at least age, gender, and level of education) reported?; (3) Was the process of classifying diagnoses upon entry into the cohort well described (use of explicit criteria, classification by consensus or application of international criteria)? Following other published reviews (Lee et al., 2008; Linde et al., 2020; Millard et al., 2013; Sangsawang, Wacharasin, & Sangsawang, 2019), a total quality percentage score was calculated for each study based on the number of “yes” responses divided by the total number of questions and given the following ratings: poor (< 50%), fair (50–69%), good (70–79%), or strong (≥ 80%).

## Results

### Selection and Inclusion of Studies

**Fig. 1:**
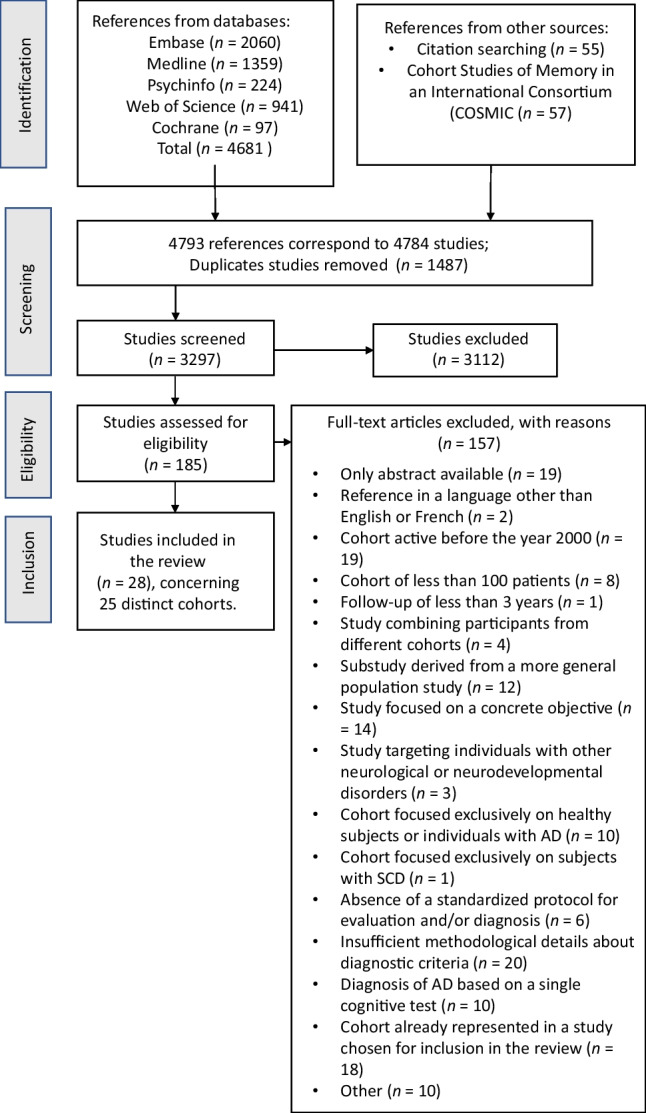
PRISMA flow diagram of the literature search and selection process

Figure 1 illustrates the literature search strategy used in this review. We identified 4793 references from different sources, including 4681 from electronic databases, and an additional 112 citations identified manually. Among the manual citations, 55 were sourced during the full-text review, and 57 were derived from the COSMIC registry. The 4793 references correspond to 4784 distinctive studies. After adjusting for duplicates, 3297 individual papers remained in the pool of eligible studies. After screening all titles and abstracts, 185 studies were retained as potentially eligible. A full-text examination led to the exclusion of 157 studies which did not meet eligibility criteria, thus leaving 28 individual studies relating to 25 distinct cohort studies for the systematic review analysis.

### Study Characteristics

The main characteristics of the 25 cohort studies included in this review are summarized in Table 1. The cohorts originated from 16 different countries, across Europe (*n* = 11), Asia (*n* = 7), America (*n* = 5), and Oceania (*n* = 2). Participants were recruited almost equally from three different sources, namely from the community, by a referral from a physician or by a combination of both. While most studies followed less than 1500 individuals, sample sizes ranged from 105 to over 10,000 participants. Follow-up evaluations were usually done within the range of 6 to 24 months.

**Table 1:** Characteristics of included cohort studies

Cohort name/country/main reference	Start date–end date or ongoing (as of March 2023) or unknown	Recruitment	Sample size (*n*)	Frequency of follow-up contact	Conversion or outcome classification*	Clinical classification disagreement handled by consensus meeting	Diagnostic confirmation
**ADNI study 1 and 2** (USA) Peterson, 2010 (Petersen et al., 2010) Aisen, 2015 (Aisen et al., 2015)	2004–ongoing	▪ Community	1171	6 months	Same as baseline	Not reported	Not reported
**AIBL study** (Australia) Ellis, 2009 (Ellis et al., 2009)	2006–ongoing	▪ Primary care physician ▪ Specialist physician ▪ Memory clinic ▪ Community	1112	18 months	Same as baseline	Yes	Imagery; Biomarker
**BioFINDER study** (Sweden) Palmqvist, 2014 (Palmqvist et al., 2014)	2010–2016, but still have some longitudinal follow-ups left. BioFINDER- 2 started in 2017 and is still recruiting	▪ Primary care physician ▪ Specialist physician ▪ Memory clinic ▪ Community	728	24 months for NC, 12 months for SCD/MCI/dementia	Same as baseline	Yes	Imagery; Biomarker
**Brazil cohort** (Brazil) Diniz, 2008 (Diniz et al., 2008) Forlenza, 2010 (Forlenza et al., 2010)	2001–2009	▪ Primary care physician ▪ Specialist physician ▪ Memory clinic ▪ Community	258	12 months	Same as baseline	Yes	Not reported
**CIMA-Q study (Canada)** Belleville, 2019 (Belleville et al., 2019)	2014–ongoing	▪ Memory clinic ▪ Community	290	12–24 months	Same as baseline	Yes	Not reported
**Compostela Aging Study** (Spain) Campos-Magdaleno, 2020 (Campos-Magdaleno et al., 2020)	2012–ongoing	▪ Primary care physician	249	18 months	Changes in psychometric scores	Yes	Not reported
**DELCODE study** (Germany) Jessen, 2018 (Jessen et al., 2018)	2014–unknown	▪ Primary care physician ▪ Community	394	12 months	Same as baseline	Not reported	Imagery; Biomarker
**German Dementia Competence Network** (Germany) Kornhuber, 2009 (Kornhuber et al., 2009)	2003–2007	▪ Specialist physician ▪ Memory clinic	2113	24–36 months	Same as baseline	Yes	Imagery; Biomarker
**Hellenic Longitudinal Investigation of Aging and Diet (HELIAD)** (Greece) Dardiotis, 2014 (Dardiotis et al., 2014)	2011–ongoing	▪ Community	1500	36 months	Same as baseline	Yes	Not reported
**HOPE study** (China) Zhu, 2021 (Zhu et al., 2021)	2020–ongoing	▪ Specialist physician	1269	12 months	Same as baseline	Yes	Imagery; Biomarker
**InveCe.AB study** (Italy) Guaita, 2013 (Guaita et al., 2013)	2010–2015	▪ Primary care physician ▪ Hospital	1321	24 months	Same as baseline	No	Biomarker
**JPSC-AD study** (Japan) Ninomiya, 2020 (Ninomiya et al., 2020)	2016–2023	▪ Community	11,410	12–24 months	Same as baseline	Yes	Imagery; Biomarker
**KBASE study** (South Korea) Lee Sohn, 2017 (Lee Sohn, 2017)	2014–ongoing	▪ Specialist physician ▪ Memory clinic ▪ Community	591	12 months	Same as baseline	Yes	Not reported
**KloSHA study** (South Korea) Park, 2007 (Park et al., 2007)	2005–2019	▪ Community	992	48 months	Same as baseline	Not reported	Imagery; Neuropathological
**Kurihara Project** (Japan) Meguro, 2012 (Meguro et al., 2012) Kumai, 2016 (Kumai et al., 2016)	2008–2013	▪ Not reported	590	Baseline and one mail follow-up between 3 and 5 years later (AD questionnaire only)	AD8 Dementia Screening Interview ≥ 2 points	Not reported	Not reported
**MEMENTO study** (France) Dufouil, 2017 (Dufouil et al., 2017)	2011–ongoing on the name MEMENTO +	▪ Specialist physician ▪ Memory clinic	2323	6 months	Changes in psychometric scores	Not reported	Not reported
**Poland cohort** (Poland) Gabryelewicz, 2007 (Gabryelewicz et al., 2007)	2001–unknown	▪ Memory clinic	105	12 months	Same as baseline	Yes	Not reported
**ROSAS study** (France) de Mauléon, 2017 (de Mauleon et al., 2017)	2014–2017	▪ Specialist physician ▪ Memory clinic	387	6 months for AD and MCI; 12 months for control	Changes in psychometric scores + clinical judgment of neuropsychiatrist	No	Not reported
**SCIENCe study** (Netherlands) Slot, 2018 (Slot et al., 2018)	2014–unknown	▪ Primary care physician ▪ Specialist physician ▪ Memory clinic	151	12 months	Same as baseline	Yes	Imagery; Biomarker
**Shanghai Aging Study** (China) Ding, 2014 (Ding et al., 2014)	2010–ongoing	▪ Community	3141	48 months	Same as baseline	Yes	Not reported
**SILCODE study** (China) Li, 2019 (Li et al., 2019)	2017–unknown	▪ Primary care physician ▪ Specialist physician ▪ Memory clinic ▪ Community	Not reported	15 months	Same as baseline	Yes	Not reported
**Sydney Memory and Ageing Study** (Australia) Sachdev, 2010 (Sachdev et al., 2010)	2005–2020	▪ Electoral roll	1037	24 months	Same as baseline	Yes	Not reported
**The Mayo Clinic Study of Aging** (USA) Roberts, 2008 (Roberts et al., 2008)	2004–unknown	▪ Community	2719	15 months	Consensus	Yes	Not reported
**Vanderbilt Memory & Aging Project** (USA) Moore, 2020 (Moore et al., 2020)	2012–unknown	▪ Primary care physician ▪ Specialist physician ▪ Memory clinic ▪ Community	335	18 months	Same as baseline	Yes	Imagery; Biomarker
**Vienna Transdanube Aging study** (Austria) Jungwirth, 2012 (Jungwirth et al., 2012)	2000–2010	▪ Community	606	30 months	Same as baseline	Not reported	Imagery; Biomarker

^*^Baseline classification: *CN*, cognitively normal; *SCD*, subjective cognitive decline; *MCI*, mild cognitive impairment; *AD*, Alzheimer disease

The diagnostic criteria used to determine conversion from one cognitive stage to another were generally the same as those used to characterize the study population at baseline. Adjudication committees were frequently used to ratify an individual’s change in clinical stage (68%). Diagnostic confirmation procedures were used in only 44% of the studies, usually through neuroimaging or biomarkers.

### Similarities and Differences in Diagnostic Criteria

Diagnostic criteria for different clinical stages of the AD continuum among cohorts are shown in Table 2 and in Supplementary material (Tables S2 A, B, C, D).

**Table 2:** Diagnostic criteria at baseline per each cohort

Cohort name/country/main reference	Cognitively normal	Subjective cognitive decline	Mild cognitive impairment	Alzheimer disease
**ADNI study 1 and 2** (USA) Peterson, 2010 (Petersen et al., 2010) Aisen, 2015 (Aisen et al., 2015)	▪ No memory complaints ▪ MMSE 24–30 (Folstein et al., 1975) ▪ CDR = 0 (Hughes et al., 1982) ▪ Delayed recall of 1 paragraph from the Logical Memory II subscale of the Wechsler Memory Scale–Revised (Online document Petersen, R. et al. (Undated). *Alzheimer’s disease neuroimaging protocol (ADNI.* Retrieved March 11, 2024, from https://adni.loni.usc.edu/wp-content/themes/freshnews-dev-v2/documents/clinical/ADNI-1_Protocol.pdf) cutoff scores = ≥ 9 for 16 years of education, ≥ 5 for 8–15 years of education, and ≥ 3 for 0–7 years of education ▪ Intact with regard to general cognition and functional performance ▪ Could not qualify for the diagnosis of dementia	▪ Cognitively normal according to study criteria ▪ Presence of memory complaints ▪ Score ≥ 16 on the first 12 questions of the Cognitive Change Index (Saykin et al., 2006)	Petersen criteria (1999) (Petersen et al., 1999)adapted: ▪ Presence of memory complaints ▪ MMSE 24–30 (Folstein et al., 1975) ▪ CDR = 0.5 (Hughes et al., 1982) ▪ Delayed recall of 1 paragraph from the Logical Memory II subscale of the Wechsler Memory Scale–Revised (Online document Petersen, R. et al. (Undated). *Alzheimer’s disease neuroimaging protocol (ADNI.* Retrieved March 11, 2024, from https://adni.loni.usc.edu/wp-content/themes/freshnews-dev-v2/documents/clinical/ADNI-1_Protocol.pdf) cutoff scores = ≤ 8 for 16 years of education, ≤ 4 for 8–15 years of education, and ≤ 2 for 0–7 years of education ▪ This group divided int Early MCI if memory function was 1.0 SD below expected adjusted norms and Late MCI if memory function was 1.5 SD below expected adjusted norms	▪ AD: NINCS-ADRDA criteria (McKhann et al., 1984) ▪ Mild AD: Presence of memory complaints MMSE 20–26 (Folstein et al., 1975) CDR = 0.5 or 1 (Hughes et al., 1982) ▪ Delayed recall of 1 paragraph from the Logical Memory II subscale of the Wechsler Memory Scale–Revised (Online document Petersen, R. et al. (Undated). *Alzheimer’s disease neuroimaging protocol (ADNI.* Retrieved March 11, 2024, from https://adni.loni.usc.edu/wp-content/themes/freshnews-dev-v2/documents/clinical/ADNI-1_Protocol.pdf) cutoff scores = ≤ 8 for 16 years of education, ≤ 4 for 8–15 years of education, and ≤ 2 for 0–7 years of education
**AIBL Study** (Australia) Ellis, 2009 (Ellis et al., 2009)	▪ No subjective memory complaint ▪ No neuropsychological performance lower or equal to 1.5 SD below the mean	Positive answer to the single question: “Do you have difficulty with your memory?”	▪ Winblad criteria (Winblad et al., 2004) ▪ A score 1.5 SD or more below the age-adjusted mean on at least one neuropsychological task of the AIBL battery (or impairment on two or more cognitive tests at a level at least 1.5 SD below the age-adjusted mean if the participant had volunteered as a healthy control)	▪ DSM-IV criteria (American Psychiatric Association, 1994) ▪ ICD- 10 criteria (Online document World Health Organization, *ICD- 10 Version:2019, Chapter V. Mental and behavioural disorders*. Retrieved March 11, 2024, from https://icd.who.int/browse10/2019/en#/F03) ▪ NINCDS-ADRDA criteria (McKhann et al., 1984)
**BioFINDER study** (Sweden) Palmqvist, 2014 (Palmqvist et al., 2014)	Not applicable	Not applicable	▪ Presence of cognitive symptoms ▪ MMSE = 24–30 (Folstein et al., 1975) ▪ Does not fulfill criteria for dementia ▪ Mean scores ≤ − 1.5 *z*-score on neuropsychological tests, but a higher performance can be classified as MCI if considered significantly below estimated premorbid level	NIA-AA criteria (McKhann et al., 2011)
**Brazil cohort** (Brazil) Diniz 2008 (Diniz et al., 2008) Forlenza, 2010 (Forlenza et al., 2010)	▪ Absence of cognitive complaints ▪ MMSE = 28–30 (Folstein et al., 1975) ▪ Do not fulfill the criteria for MCI or any dementia	▪ Presence of cognitive symptoms ▪ MMSE = 24–30 (Folstein et al., 1975) ▪ Do not fulfill the criteria for any dementia ▪ No significant impairment in any neuropsychological tests results	Petersen criteria (1999) (Petersen et al., 1999)	NINCDS-ADRDA criteria (McKhann et al., 1984)
**CIMA-Q study (Canada)** Belleville, 2019 (Belleville et al., 2019)	▪ No memory complaints, or presence of complaints without feeling worried about them ▪ MoCA ≥ 26/30 ▪ Delayed recall of 1 paragraph from the Logical Memory II subscale of the Wechsler Memory Scale–Revised (Online document Petersen, R. et al. (Undated). *Alzheimer’s disease neuroimaging protocol (ADNI.* Retrieved March 11, 2024, from https://adni.loni.usc.edu/wp-content/themes/freshnews-dev-v2/documents/clinical/ADNI-1_Protocol.pdf) cutoff scores = ≥ 9 for 16 years of education, ≥ 5 for 8–15 years of education, and ≥ 3 for 0–7 years of education ▪ CDR = 0 (Hughes et al., 1982)	Jessen criteria (Jessen et al., 2014)	▪ NIA-AA criteria (Albert et al., 2011) ▪ Jessen a or b criteria (Jessen et al., 2014) ▪ CDR = 0.5 or 1 (Hughes et al., 1982) ▪ Early MCI: ▪ MoCA between 20 and 26 ▪ Wechsler Memory Scale-Revised (Wechsler, 1987) cutoff scores 9–11 for 16 years of education, scores 5–9 for 8–15 years of education, and scores 3–6 for 0–7 years of education ▪ Late MCI: ▪ MoCA between 20 and 25 ▪ Wechsler Memory Scale-Revised (Wechsler, 1987) cutoff scores = ≤ 8 for 16 years of education, ≤ 4 for 8–15 years of education, and ≤ 2 for 0–7 years of education	▪ NIA-AA criteria (McKhann et al., 2011) ▪ Jessen a or b criteria (Jessen et al., 2014) ▪ MoCA between 13 and 25 ▪ Wechsler Memory Scale-Revised [50] cutoff scores = ≤ 8 for 16 years of education, ≤ 4 for 8–15 years of education, and ≤ 2 for 0–7 years of education ▪ CDR = 0.5 or 1 (Hughes et al., 1982)
**Compostela Aging Study** (Spain) Campos-Magdaleno, 2020 (Campos-Magdaleno et al., 2020)	Not reported	▪ Subjective cognitive complains, confirmed by the SMCQ questionnaire (Benedet & Seisdedos, 1996) ▪ Normal cognitive performance according to adjusted norms in CAMCOG-R (Huppert et al., 1996) and the California Verbal Learning Test (Delis et al., 1987)	NIA-AA criteria (Albert et al., 2011)	▪ DSM-IV criteria (American Psychiatric Association, 1994) ▪ NINCDS-ADRDA criteria (McKhann et al., 1984)
**DELCODE study** (Germany) Jessen, 2018 (Jessen et al., 2018)	▪ Absence of subjectively reported decline in cognitive functioning with concerns ▪ Test performance of better than − 1.5 SD below the norm on all subtests of the CERAD neuropsychological battery	Jessen criteria (Jessen et al., 2014)	NIA-AA criteria (Albert et al., 2011)	NIA-AA criteria (McKhann et al., 2011)
**German Dementia Competence Network** (Germany) Kornhuber, 2009 (Kornhuber et al., 2009)	▪ No MCI at baseline (CDR = 0) (Hughes et al., 1982) ▪ All cognitive tests within the normal range	Not applicable	▪ Complaints of cognitive deficit in daily living ▪ Objectified decline of cognitive abilities (more than 1 SD) in at least 1 cognitive domain, as evidenced by standardized neuropsychological tests ▪ Bayer Activities Daily Living scale < 4 (Hindmarch et al., 1998)	NINCDS-ADRDA criteria (McKhann et al., 1984)
**Hellenic Longitudinal Investigation of Aging and Diet (HELIAD)** (Greece) Dardiotis, 2014 (Dardiotis et al., 2014)	Not applicable	Not applicable	Petersen 2001 (Petersen et al., 2001)	▪ Dementia: DSM-IV-TR criteria (American Psychiatric Association, 2000) ▪ AD: NINCDS/ADRDA criteria (McKhann et al., 1984)
**HOPE study** (China) Zhu, 2021 (Zhu et al., 2021)	Not reported	Not applicable	NIA-AA criteria (Albert et al., 2011)	NIA-AA criteria (McKhann et al., 2011)
**InveCe.AB study** (Italy) Guaita, 2013 (Guaita et al., 2013)	Not reported	Not applicable	▪ Petersen criteria (2004) (Petersen, 2004) ▪ CIND criteria (Chertkow et al., 2008) if self-report of cognitive problems was not available	▪ DSM IV-TR criteria (American Psychiatric Association, 2000) ▪ NINCDS-ADRDA criteria (McKhann et al., 1984)
**JPSC-AD study** (Japan) Ninomiya, 2020 (Ninomiya et al., 2020)	▪ MMSE > 26 (Folstein et al., 1975) or ▪ MMSE ≤ 26 but normal performance on the delayed recall test of the Wechsler Memory Scale Revised (Online document Petersen, R. et al. (Undated). *Alzheimer’s disease neuroimaging protocol (ADNI.* Retrieved March 11, 2024, from https://adni.loni.usc.edu/wp-content/themes/freshnews-dev-v2/documents/clinical/ADNI-1_Protocol.pdf) and the Pareidolia test (Mamiya et al., 2016)	Not applicable	Petersen criteria (2001) (Petersen et al., 2001)	▪ DSM-III R criteria (American Psychiatric Association, 1987) ▪ NINCDS-ADRDA criteria (McKhann et al., 1984)
**KBASE study** (South Korea) Lee Sohn, 2017 (Lee Sohn, 2017)	▪ 20–54 years of age (young adult group) or 55–90 years of age (elderly adult group) ▪ CDR = 0 (Hughes et al., 1982) ▪ No diagnosis of MCI or dementia	Not applicable	▪ NIA-AA criteria (Albert et al., 2011) ▪ CDR = 0.5 (Hughes et al., 1982)	▪ DSM-IV-TR IV criteria (American Psychiatric Association, 2000) ▪ NIA-AA criteria (McKhann et al., 2011) ▪ CDR = 0.5 or 1 (Hughes et al., 1982)
**KLoSHA study** (Korea) Park, 2007 (Park et al., 2007)	Not applicable	Not applicable	International Working Group Criteria for Mild Cognitive Impairment (Winblad et al., 2004)	▪ DSM-IV criteria (American Psychiatric Association, 1994) ▪ NINCDS-ADRDA criteria (McKhann et al., 1984)
**Kurihara Project** (Japan) Meguro, 2012 (Meguro et al., 2012) Kumai, 2016 (Kumai et al., 2016)	CDR = 0	Not applicable	▪ CDR 0.5 diagnosis of “questionable dementia” ▪ Alzheimer’s disease criteria: CDR memory domain 0.5 (no formal memory tests carried out except MMSE); no neurological symptoms; with or without executive dysfunction (TMT-A and B); no cerebrovascular disease (clinical or MRI)	▪ DSM IV criteria (American Psychiatric Association, 1994) + NINCDS-ADRDA criteria (McKhann et al., 1984) (prevalence) ▪ CDR = 1 (Hughes et al., 1982) + AD8 Dementia Screening Interview ≥ 2 (incidence)
**MEMENTO study** (France) Dufouil, 2017 (Dufouil et al., 2017)	Not applicable	▪ Subjective cognitive complaints (visual analogue scales) ▪ Criteria for MCI not met	▪ Performance of 1 SD worse than the subject’s own age, sex, and education-level group mean in one or more cognitive domains ▪ CDR ≤ 0.5 (Hughes et al., 1982)	Not applicable
**Poland cohort** (Poland) Gabryelewicz, 2007 (Gabryelewicz et al., 2007)	Not applicable	Not applicable	▪ Petersen criteria (1997) (Petersen et al., 1997) ▪ CDR = 0.5 (Hughes et al., 1982)	DSM-III R criteria (American Psychiatric Association, 1987)
**ROSAS study** (France) de Mauléon, 2017 (de Mauleon et al., 2017)	▪ Absence of memory impairment on the *Rey Auditory Verbal Learning Test (*Rey, 1964*)* ▪ MMSE ≥ 26 (Folstein et al., 1975) ▪ CDR = 0 (Hughes et al., 1982)	Not applicable	▪ DSM-IV-RT criteria of Alzheimer’s dementia not met (American Psychiatric Association, 2000) ▪ Rey Auditory Verbal Learning test (Rey, 1964) < 1 SD of age-adjusted mean ▪ MMSE ≥ 24 (Folstein et al., 1975) ▪ CDR = 0.5 (Hughes et al., 1982)	▪ DSM-IV-TR criteria (American Psychiatric Association, 2000) ▪ MMSE = 12–26 (Folstein et al., 1975) ▪ CDR ≥ 0.5 (Hughes et al., 1982)
**SCIENCe study** (Netherlands) Slot, 2018 (Slot et al., 2018)	Not reported	▪ Cognitive complaints and normal cognition (clinical diagnosis) ▪ No diagnosis of MCI or dementia, or any other disease known to cause memory complaints. “SCD Plus” group categorized using Jessen criteria (Jessen et al., 2014)	Clinical diagnosis (memory clinic)	Clinical diagnosis (memory clinic)
**Shanghai Aging Study** (China) Ding, 2014 (Ding et al., 2014)	Not reported	Not applicable	Petersen criteria (2004) (Petersen, 2004)	▪ DSM IV criteria (American Psychiatric Association, 1994) ▪ NINCDS-ADRDA criteria (McKhann et al., 1984)
**SILCODE study** (China) Li, 2019 (Li et al., 2019)	Not applicable	Jessen criteria (Jessen et al., 2014)	Jak and Bondi MCI actuarial criteria (Bondi et al., 2014)	▪ DSM- 5 criteria (American Psychiatric Association, 2013) ▪ NIA-AA criteria (McKhann et al., 2011) ▪ CDR ≥ 1 (Hughes et al., 1982)
**Sydney Memory and Ageing Study** (Australia) Sachdev, 2010 (Sachdev et al., 2010)	▪ Performance on all test measures above − 1.5 SDs compared to normative values ▪ Not demented ▪ Normal function or minimal impairment (score < 3.0 on the Bayer Activities of Daily Living scale (Hindmarch et al., 1998)) ▪ Cognitive complaint was allowed	Not applicable	Winblad criteria (Winblad et al., 2004)	DSM-IV criteria (American Psychiatric Association, 1994)
**The Mayo Clinic Study of Aging** (USA) Roberts, 2008 (Roberts et al., 2008)	▪ No cognitive impairment according to WAIS-R (Wechsler, 1981), WMS-R (Wechsler, 1987) and Auditory Verbal Learning Test (Ivnik et al., 1992) norms ▪ Petersen criteria for MCI (2004) (Petersen, 2004) not met ▪ CDR = 0 (Hughes et al., 1982)	Not applicable	▪ Petersen criteria (2004) (Petersen, 2004) ▪ CDR = 0 or 0.5 (Hughes et al., 1982)	▪ DSM-IV criteria (American Psychiatric Association, 1994) ▪ CDR = ≥ 0.5 (Hughes et al., 1982) (if CDR = 0.5, diagnosis of dementia was confirmed by consensus) ▪ Impaired functional status
**Vanderbilt Memory & Aging Project** (USA) Moore, 2020 (Moore et al., 2020)	▪ CDR = 0 (Hughes et al., 1982) ▪ No evidence of impairment on cognitive tests (no scores outside of 1.5 SD of the age-adjusted mean)	Not applicable	NIA-AA criteria (Albert et al., 2011)	Not reported
**Vienna Transdanube Aging *study*** (Austria) Jungwirth, 2012 (Jungwirth et al., 2012)	Performances within the 1.5 SD range in all psychometric tests	Not applicable	Petersen criteria (2004) (Petersen, 2004)	NINCDS-ADRDA criteria (McKhann et al., 1984)

*SD*, standard deviation; *SCD*, subjective cognitive decline; *MCI*, mild cognitive impairment; *AD*, Alzheimer disease; *CDR*, Clinical Dementia Rating; *MMSE*, Mini-Mental State Examination; *CERAD*, Consortium to Establish a Registry for Alzheimer’s Disease neuropsychological test battery (www.memoryclinic.ch); *SMCQ*, Subjective Memory Complaints Questionnaire; *CAMCOG-R*, Spanish adapted version of the Cambridge Cognitive Examination; *WAIS-R*, Wechsler Adult Intelligence Scale-Revised; *WMS-R*, Wechsler Memory Scale-Revised; *SRT*, Selective Reminding Test, 12-item, 6-trial version

The diagnosis of dementia was determined in 23 of the 25 cohorts (92%), since one study was not concerned with following individuals with dementia (Dufouil et al., 2017), and another one (Moore et al., 2020) did not report its classification strategy for this stage of the disease. Of the 23 cohorts, four (de Mauleon et al., 2017; Gabryelewicz et al., 2007; Roberts et al., 2008; Sachdev et al., 2010) identified only the presence of dementia, but not AD, using DSM criteria; two of these (de Mauleon et al., 2017; Roberts et al., 2008) relied on CDR scores as well. The remaining 19 cohorts used specific criteria to classify their individuals with AD dementia. Ten (Campos-Magdaleno et al., 2020; Dardiotis et al., 2014; Ding et al., 2014; Ellis et al., 2009; Guaita et al., 2013; Kumai et al., 2016; Lee Sohn, 2017; Li et al., 2019; Meguro et al., 2012; Ninomiya et al., 2020; Park et al., 2007) of them used a two-step process: initially identifying dementia based on DSM criteria, followed by the application of NINCDS-ADRDA (McKhann et al., 1984) in eight cohorts and NIA-AA criteria (McKhann et al., 2011) in two cohorts (Lee Sohn, 2017; Li et al., 2019). In contrast, eight cohorts did not use the DSM criteria at all: four of them (Aisen et al., 2015; Diniz et al., 2008; Forlenza et al., 2010; Jungwirth et al., 2012; Kornhuber et al., 2009; Petersen et al., 2010) identified AD dementia according to NINCDS-ADRDA criteria only (McKhann et al., 1984); the other four (Belleville et al., 2019; Jessen et al., 2018; Palmqvist et al., 2014; Zhu et al., 2021) used the NIA-AA criteria (McKhann et al., 2011) exclusively. One study classified their participants solely according to clinical diagnosis (Slot et al., 2018).

All 25 cohorts included in this review identified the presence of mild cognitive impairment (MCI). Nineteen of these used internationally recognized diagnostic criteria. Nine out of the 19 studies (Aisen et al., 2015; Dardiotis et al., 2014; Ding et al., 2014; Diniz et al., 2008; Forlenza et al., 2010; Gabryelewicz et al., 2007; Guaita et al., 2013; Jungwirth et al., 2012; Ninomiya et al., 2020; Petersen et al., 2010; Roberts et al., 2008) used the Petersen 1999 (Petersen et al., 1999), 2001 (Petersen et al., 2001), or 2004 (Petersen, 2004) criteria; one of them (Guaita et al., 2013) also used the CIND criteria if self-report was not available, and two studies (Gabryelewicz et al., 2007; Roberts et al., 2008) also used CDR total scores. Six of the aforementioned 19 studies (Belleville et al., 2019; Campos-Magdaleno et al., 2020; Jessen et al., 2018; Lee Sohn, 2017; Moore et al., 2020; Zhu et al., 2021) used the NIA-AA criteria for MCI (Albert et al., 2011), one of which (Lee Sohn, 2017) also used CDR total scores. Three of the 19 studies (Ellis et al., 2009; Park et al., 2007; Sachdev et al., 2010) used the Winblad/IWG criteria for MCI (Winblad et al., 2004), and one of them (Ellis et al., 2009) required abnormal performance in at least one task of their cognitive battery. Another study (Li et al., 2019) used the MCI actuarial criteria proposed by Jak and Bondi (Bondi et al., 2014). Of the 25 cohorts included in this review, five (de Mauleon et al., 2017; Dufouil et al., 2017; Kornhuber et al., 2009; Kumai et al., 2016; Meguro et al., 2012; Palmqvist et al., 2014) developed their own MCI criteria and one study (Slot et al., 2018) relied on clinical diagnosis only.

Study participants were classified as having subjective cognitive decline (SCD) in nine of the 25 cohorts (36%) (Aisen et al., 2015; Belleville et al., 2019; Campos-Magdaleno et al., 2020; Dufouil et al., 2017; Ellis et al., 2009; Jessen et al., 2018; Li et al., 2019; Palmqvist et al., 2014; Petersen et al., 2010; Slot et al., 2018). Four of these cohorts (Belleville et al., 2019; Jessen et al., 2018; Li et al., 2019; Slot et al., 2018) used the Jessen criteria (Jessen et al., 2014), with one study (Slot et al., 2018) relying on clinical diagnosis for SCD categorization and used the Jessen criteria specifically to identify a subgroup labeled as “SCD Plus.” The other five cohorts (Aisen et al., 2015; Campos-Magdaleno et al., 2020; Dufouil et al., 2017; Ellis et al., 2009; Palmqvist et al., 2014; Petersen et al., 2010) relied on prespecified diagnostic criteria created for each of their studies, relying on open questions, questionnaires, scales, normal scores on cognitive tests, or a combination of these, as seen on Table 2.

Twenty of the 25 cohorts (80%) also followed individuals considered to have normal cognition (NC) (Aisen et al., 2015; Belleville et al., 2019; Campos-Magdaleno et al., 2020; de Mauleon et al., 2017; Ding et al., 2014; Diniz et al., 2008; Ellis et al., 2009; Forlenza et al., 2010; Guaita et al., 2013; Jessen et al., 2018; Jungwirth et al., 2012; Kornhuber et al., 2009; Kumai et al., 2016; Lee Sohn, 2017; Meguro et al., 2012; Moore et al., 2020; Ninomiya et al., 2020; Palmqvist et al., 2014; Petersen et al., 2010; Roberts et al., 2008; Sachdev et al., 2010; Slot et al., 2018; Zhu et al., 2021). Although no diagnostic criteria for this group were the same in any of the studies, seven (Aisen et al., 2015; Belleville et al., 2019; de Mauleon et al., 2017; Kumai et al., 2016; Lee Sohn, 2017; Meguro et al., 2012; Moore et al., 2020; Petersen et al., 2010; Roberts et al., 2008) used a total CDR score of 0 as part of their criteria; five used screening assessment instruments for global cognition within the normal ranges (MMSE in four studies Aisen et al., 2015; de Mauleon et al., 2017; Ninomiya et al., 2020; Palmqvist et al., 2014; Petersen et al., 2010); one study used the MoCA (Belleville et al., 2019)); twelve studies (Aisen et al., 2015; Belleville et al., 2019; de Mauleon et al., 2017; Diniz et al., 2008; Ellis et al., 2009; Forlenza et al., 2010; Jessen et al., 2018; Jungwirth et al., 2012; Kornhuber et al., 2009; Moore et al., 2020; Ninomiya et al., 2020; Petersen et al., 2010; Roberts et al., 2008; Sachdev et al., 2010) relied on cognitive tests or batteries within the normal range or within 1.5 SD of the age-adjusted mean. Five studies (Campos-Magdaleno et al., 2020; Ding et al., 2014; Guaita et al., 2013; Slot et al., 2018; Zhu et al., 2021) did not report any specific criteria to define this group.

### Summary of Quality Assessment

The risk of bias assessment results for each threat to the internal validity and the results of the global quality assessment range of the 25 included cohort studies are presented in Table 3. According to the modified NIH quality assessment tool, 19 studies revealed a lower risk of bias since they were rated as strong (*n* = 3) or good (*n* = 16) quality. In contrast, six studies displayed a relatively greater risk of bias since they were rated as being of fair (*n* = 5) or poor (*n* = 1) quality. The most common limitations were related to not reported information about the blinding assessment of intermediate cognitive conditions or the outcome (Q12), the percentage of losses at follow-up (Q13), and the consideration of potential confounders (Q14). Moreover, information on the participation rate of eligible persons (Q3) was missing from 48% of the articles and the sample size used was not justified (Q5) in 80% of them. However, Table 3 shows that the method of recruitment (Q15), the characteristics of the participants (Q16), and the process of classifying diagnoses at baseline (Q17) were usually well described and reported in the selected articles.

**Table 3:**
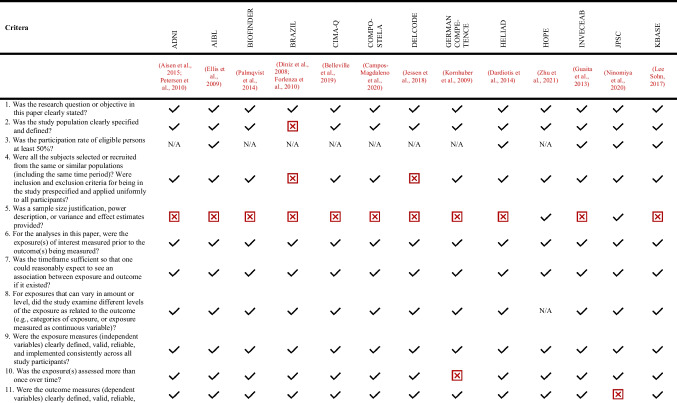

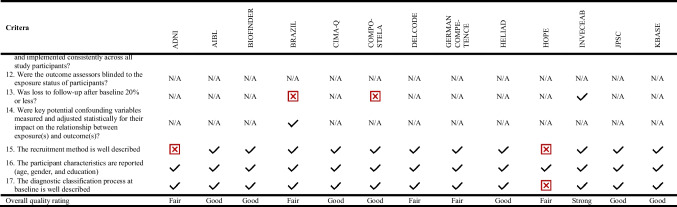

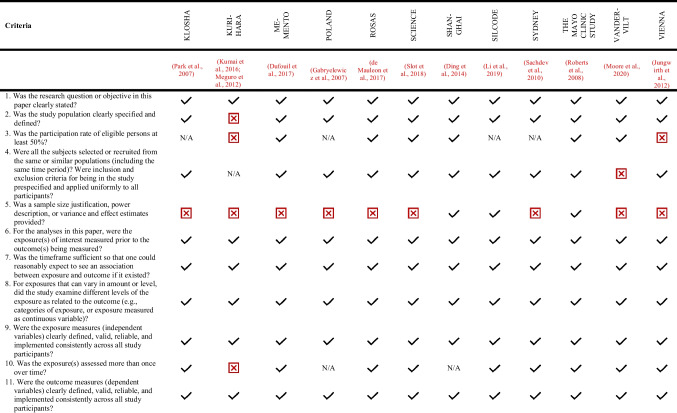

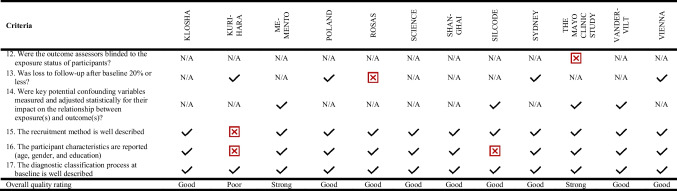
Evaluation of bias according to the modify National Institutes of Health (NIH) quality assessment tool for observational cohort and cross-sectional studies

= yes;  = no; *N/A*, not available, not assessed, cannot determined, or not reported in the article consulted

Percentage score of relevant criteria met by each study: strong (≥ 80%), good (70–79%), fair (50–69%), or poor (≥ 50%)

## Discussion

The rigor in the clinical categorization of all individuals participating in prospective, longitudinal, population-based cohorts concerned with the clinical AD continuum is an essential element of study design. This greatly helps to obtain the most accurate data at each stage of the disease. The multiplicity of diagnostic criteria available and the use of different versions modified over the years could hinder the extrapolation and comparison of results with other longitudinal AD cohorts, and greatly limit collaboration efforts. We carried out a systematic review of the published literature in recent years with the aim of finding out the potential scope of this problem, document it and consolidate relevant findings.

Of the 23 cohorts that have focused on the identification and follow-up of people in the Alzheimer’s disease continuum, we found that 21 based the core of their classification process either on the DSM criteria (13/23) or the NINCDS-ADRDA or NIA-AA criteria (17/23). It must be noted that, while not interchangeable, the NIA-AA criteria are considered to be an evolution of the NICNCDS-ADRDA criteria. Only two cohorts dispensed with either set of criteria, opting to diagnose AD by clinical diagnosis (SCiENCe cohort (Slot et al., 2018)) or relying on the CDR protocol (KURIHARA cohort Kumai et al., 2016; Meguro et al., 2012)).

All 25 cohorts had classification strategies to identify individuals with MCI. While convergence of the criteria was not as strong as for AD, 15 out of the 25 relied on the Petersen or NIA-AA criteria for MCI diagnosis; the NIA-AA criteria can be considered to be an evolution of the Petersen criteria. Eight cohorts used the CDR protocol as a main component of their MCI classification, five of which were also part of the aforementioned 15 studies that relied on the Petersen/NIA-AA criteria for classification purposes. Three other cohorts mainly used the Winblad-IWG criteria to classify their MCI subjects. There is little convergence between the rest of the studies.

Nine of the 25 cohorts were concerned with individuals with Subjective Cognitive Decline. Classification criteria for SCD were less consistent. Of these, only four of them used a specific set of internationally accepted diagnostic criteria (Jessen et al., 2014). This is explained by the fact that the “Jessen criteria” were published in 2014, and all five cohorts that did not use them had started their recruitment before this year. While the concept and definition of SCD will certainly evolve over time, the use of a common set of standardized criteria would certainly help to facilitate its study.

Our systematic review showed that the highest variability in the classification of study participants was seen in the control, or cognitively unimpaired group. There is no internationally accepted set of clinical criteria for “cognitively normal” individuals. These tend to be defined by the absence of impairment using a wide-ranging set of indicators, which can range from the absence of complaints to normal scores in short screening instruments or in a wide variety of cognitive tests. As the emergence of SCD as a recognized preclinical entity has shown, a vague defined definition of “normality” could hinder the study of the earliest clinical changes of AD. The development of a minimal, standardized, and operationalized definition for “cognitively normal” individuals in population-based cohort studies would benefit future research, particularly as focus shifts towards identifying the earliest stages of AD. A standardized, working definition of “normal cognition” will still be useful in the age of AD diagnosis carried out with the aid of biomarkers. For example, the International Working Group (Dubois et al., 2024) introduced the concept of “Asymptomatic at Risk for Alzheimer’s disease” for those individuals without cognitive impairment that have a positive biomarker profile for AD. This group of individuals merit further study and longitudinal cohorts will play an important role in better understanding their disease trajectory.

Interestingly, only five of the 25 cohorts studied were concerned with following the full spectrum of the preclinical AD continuum, from normal controls to SCD to MCI to AD (ADNI Aisen et al., 2015; Petersen et al., 2010), AIBL (Ellis et al., 2009), BioFINDER (Palmqvist et al., 2014), CIMA-Q (Belleville et al., 2019), and DELCODE (Jessen et al., 2018)). The CIMA-Q cohort (Belleville et al., 2019) also compares favorably since, by design, it uses the most frequent standardized criteria for each stage of the disease, which is particularly useful for comparison and scientific collaboration purposes.

The methodological quality of all selected publications was evaluated as part of the systematic review process. In this case, we found that more than three-quarters of the selected publications were of good to strong methodological quality, as based on a risk of bias assessment, and the others were categorized as having a fair quality rating. These findings lend credibility and robustness to the aforementioned results, as the selected studies were shown to have a high internal validity. The selected publications from one cohort (KURIHARA Kumai et al., 2016; Meguro et al., 2012)) did not report or discuss the information evaluated by the NIH quality assessment tool in their text, so it was found to be of poor methodological quality. It must be emphasized that this type of assessment is concerned only with the information found in the published articles selected for review, and it makes no assumption on the scientific or methodological quality of the source studies themselves, or the findings derived from any research carried out using data from said study cohorts.

One must acknowledge some limitations that could affect the results of this review. Multiple databases were consulted and we tried to be as inclusive and wide-ranging in our definitions as possible without affecting the scope of the objectives. Still, some relevant cohorts, particularly those comprised exclusively of individuals without cognitive impairment, and those active before the year 2000, would have been overlooked. This selection was intentional, since one of our main interests was the prodromal stages of the AD continuum (SCD and MCI) and the concept of mild cognitive impairment was still evolving in the late 1990 s. We also decided to exclude any cohort that had not been conceptualized with the study of AD as its main focus, which is one of the main reasons why some renowned and important cohort studies of the past years might not appear on our list. We are cognizant to the fact that limiting our search to studies in English or in French, languages in which the reviewers are sufficiently proficient to analyze the published texts, could have caused the omission of a few relevant cohorts. Regarding the risk of bias assessment, our research group made a reasoned choice among the available quality assessment tools. We also added three criteria to better adapt it for our analyses, since our review did not directly address a standard aspect of cohort studies and we felt that some relevant parameters were not directly covered by it. The team’s epidemiologist noted a significant agreement between his independent review and those of the two coders without this being quantified. The discrepancies were mainly due to different applications of the certain response options such as “the information was not assessed,” “could not be determined,” or “was not reported in the article.” In the end, all these responses were grouped into the same category, in order to minimize the impact of these discrepancies. We also recognize the potential limit of not having cross-verified the extracted data. Finally, it must be acknowledged that information was derived from studies published at a specific date, and any subsequent changes in a cohort’s methodology or choice of diagnostic criteria published later on during the course of the study could have been missed. On the other hand, we consider that some of the strengths of our review were the choice to limit our research to recent and active cohorts, the focus on AD and its prodromal stages, the wide-encompassing search strategy, and the fact that we tried to contact the main authors of each study to gather additional information relevant to our review when needed.

The use of PET scan and/or CSF biomarkers is now an essential part of Alzheimer research, but its use in cohort studies still poses a significant challenge because of limited accessibility and costs. It must be noted that, while some of the studies identified by our systematic review collect and analyze fluid and imaging biomarkers as part of their research objectives, none of them used these as part of their initial classification system. Future cohort studies should make a concerted effort to include biomarkers as part of their participant selection process and classification.

Over the next few years, blood-based biomarkers for AD will increasingly become available for research and clinical purposes. They will be of particular interest for epidemiologic and cohort studies because of their ease of administration, low logistical burden, low risk to obtain samples, and reduced cost. They could ensure a more accurate classification of their participants, as well as facilitate the inclusion of historically underrepresented groups (Hayes-Larson et al., 2024). These biomarkers can be obtained from stored samples, so cohort studies currently in the planning stages that still have limited or no access to CSF biomarkers or PET imaging would be well served to factor in a biobank for future diagnostic purposes. Once the current issues concerning validity, reliability, generalizability, and dynamic range are fully resolved, studies with banked blood samples could also be used to reclassify their participants according to a biological-based definition of AD. In the meantime, we support the notion of adding the term “clinical syndrome” whenever a study subject is diagnosed with AD or any other stage in the Alzheimer’s clinical continuum without biological (i.e., biomarker-based) or neuropathological confirmation.

The emergence of biomarkers as a central element in the diagnosis of AD will allows for a more accurate identification, but the debate around its definition, according to different recently published criteria, continues to this day (Petersen et al., 2024). While the field of study of AD and its prodromal and preclinical stages is shifting towards a biomarker-based diagnosis, the use of clinical diagnostic criteria for the different clinical stages of AD will still be a useful tool, especially for large cohorts and studies carried out in developing countries. This study adds to the growing literature that draws attention to the fact that the existence of multiple diagnostic criteria could affect the comparability and the generalizability of results. We would propose the development of a single, internationally accepted set of diagnostic criteria for each stage of the Alzheimer’s disease clinical continuum, specifically designed for epidemiological and cohort studies, which could then be used and operationalized in a standardized and systematic manner. This would be especially relevant for MCI and SCD where there is much less consensus when it comes to the operationalization of classification criteria, and for normal cognition in particular where no formal criteria exist.

Adopting a common set of clinical diagnostic criteria for cohort studies, rather than relying on criteria developed specifically for any given study, will be even more relevant in this era of Big Data, open science and development of electronic, collaborative research hubs that regroup multiple regional, national, and international cohorts, such as the UK Dementia Research Platform (https://www.dementiasplatform.uk/) or the University of New-South Wales’ worldwide COSMIC consortium (https://cheba.unsw.edu.au/consortia/cosmic). This would also facilitate international cooperation, assist with data harmonization across different study cohorts, and simplify result comparisons and reproducibility across different study groups and populations.

## Conclusion

This systematic review, covering 25 current or recently active cohorts focusing on the AD continuum, reveals a consensus in the choice of diagnostic criteria, particularly for later stages such as AD dementia. However, this consensus is not as strong for earlier stages like MCI and SCD, as well as in defining normal controls. This review could be of interest in the planning of future populational studies centered on the clinical stages of AD, facilitating the comparison between cohorts and the generalization of their results, as well as aiding in the collaboration efforts between different study groups.

## Supplementary Information

Below is the link to the electronic supplementary material.

Supplementary file1 (PDF 181 KB)

## Data Availability

Data sharing is not applicable to this article as no new data were created or analyzed in this systematic review study other than bibliographic information on publications that is already provided in the article and supplementary material.
