# Supplementary material for: A Comparison of Clinical Diagnostic Classification Criteria Used in Longitudinal Cohort Studies of the Alzheimer’s Disease Continuum: A Systematic Review
Source: Neuropsychol Rev. 2025 May 9;36(1):3–24. doi: 10.1007/s11065-025-09663-9 (PMC13279743; doi:10.1007/s11065-025-09663-9)
Supplement: Supplementary file 1 — Supplementary file1 (PDF 181 KB) [file 11065_2025_9663_MOESM1_ESM.pdf]

SUPPLEMENTARY MATERIAL

Appendix A. Search strategy for each database

MEDLINE database

aging/ or middle aged/ or exp aged/ or (elder* or ageing or aging or senior* or old* or aged* or geriatric* or gerontolog* or "late onset" or "late-onset").tw,kf.

alzheimer disease/ or (AD or Alzheimer*).tw,kf.

cohort studies/ or follow up studies/ or prospective studies/ or longitudinal studies/ or (cohort stud* or prospective stud* or follow up stud* or longitudinal stud* or prospective cohort stud*).tw,kf.
((clinic* adj2 (criteria* or diagnos* or assess* or evaluat* or classif*)) or ((Consensus or expert* or panel or special*) adj2 (diagnos* or assess* or evaluat* or classif*)) or ((diagnos* or cognitive or classif*) adj2 (measure* or test* or instrument* or eval* or criteria* or indicator* or tool* or scale* or assess* or questionnaire* or guideline*))).tw,kf.
memory disorders/ or cognitive dysfunction/ or (prodromal or preclinical or pre-clinical or predementia or pre-dementia or early stage or questionable Alzheimer or questionable AD or MCI or SCD or (mild cognitive adj2 (impairment or decline or deficit or disorder)) or (subject* adj2 (cognitive decline or memory complaint or cognitive impairment))).tw,kf.

EMBASE database

aging/ or middle aged/ or exp aged/ or (elder* or ageing or aging or senior* or old* or aged* or geriatric* or gerontolog* or "late onset" or "late-onset").tw,kf.

alzheimer disease/ or (AD or Alzheimer*).tw,kf.

cohort studies/ or follow up studies/ or prospective studies/ or longitudinal studies/ or (cohort stud* or prospective stud* or follow up stud* or longitudinal stud* or prospective cohort stud*).tw,kf.

((clinic* adj2 (criteria* or diagnos* or assess* or evaluat* or classif*)) or ((Consensus or expert* or panel or special*) adj2 (diagnos* or assess* or evaluat* or classif*)) or ((diagnos* or cognitive or classif*) adj2 (measure* or test* or instrument* or eval* or criteria* or indicator* or tool* or scale* or assess* or questionnaire* or guideline*))).tw,kf.

mild cognitive impairment/ or memory disorders/ or (prodromal or preclinical or pre-clinical or predementia or pre-dementia or early stage or questionable Alzheimer or questionable AD or MCI or SCD or (mild cognitive adj2 (impairment or decline or deficit or disorder)) or (subject* adj2 (cognitive decline or memory complaint or cognitive impairment))).tw,kf.

PsycINFO database

aging/ or middle adulthood/ or older adulthood/ or (elder* or ageing or aging or senior* or old* or aged* or geriatric* or gerontolog* or "late onset" or "late-onset").tw.

alzheimer disease/ or (AD or Alzheimer*).tw.

cohort analysis/ or followup studies/ or prospective studies/ or longitudinal studies/ or (cohort stud* or prospective stud* or follow up stud* or longitudinal stud* or prospective cohort stud*).tw.

((clinic* adj2 (criteria* or diagnos* or assess* or evaluat* or classif*)) or ((Consensus or expert* or panel or special*) adj2 (diagnos* or assess* or evaluat* or classif*)) or ((diagnos* or cognitive or classif*) adj2 (measure* or test* or instrument* or eval* or criteria* or indicator* or tool* or scale* or assess* or questionnaire* or guideline*))).tw.

mild cognitive impairment/ or memory disorders/ or (prodromal or preclinical or pre-clinical or predementia or pre-dementia or early stage or questionable Alzheimer or questionable AD or MCI or SCD or (mild cognitive adj2 (impairment or decline or deficit or disorder)) or (subject* adj2 (cognitive decline or memory complaint or cognitive impairment))).tw.

EBM Reviews - Cochrane Central Register of Controlled Trials, EBM Reviews - Cochrane Clinical Answers

aging/ or middle aged/ or exp aged/ or (elder* or ageing or aging or senior* or old* or aged* or geriatric* or gerontolog* or "late onset" or "late-onset").tw,kw.

alzheimer disease/ or (AD or Alzheimer*).tw,kw.

cohort studies/ or follow up studies/ or prospective studies/ or longitudinal studies/ or (cohort stud* or prospective stud* or follow up stud* or longitudinal stud* or prospective cohort stud*).tw,kw.

((clinic* adj2 (criteria* or diagnos* or assess* or evaluat* or classif*)) or ((Consensus or expert* or panel or special*) adj2 (diagnos* or assess* or evaluat* or classif*)) or ((diagnos* or cognitive or classif*) adj2 (measure* or test* or instrument* or eval* or criteria* or indicator* or tool* or scale* or assess* or questionnaire* or guideline*))).tw,kw.

memory disorders/ or cognitive dysfunction/ or (prodromal or preclinical or pre-clinical or predementia or pre-dementia or early stage or questionable Alzheimer or questionable AD or MCI or SCD or (mild cognitive adj2 (impairment or decline or deficit or disorder)) or (subject* adj2 (cognitive decline or memory complaint or cognitive impairment))).tw,kw.

Web of science

(elder* or ageing or aging or senior* or old* or aged* or geriatric* or gerontolog* or "late onset" or "late-onset")

(AD or Alzheimer*)

("cohort stud*" or "prospective stud*" or "follow up stud*" or "longitudinal stud*" or "prospective cohort stud*")

((clinic* NEAR/2 (criteria* or diagnos* or assess* or evaluat* or classif*)) or ((Consensus or expert* or panel or special*) NEAR/2 (diagnos* or assess* or evaluat* or classif*)) or ((diagnos* or cognitive or classif*) NEAR/2 (measure* or test* or instrument* or eval* or criteria* or indicator* or tool* or scale* or assess* or questionnaire* or guideline*)))

(prodromal or preclinical or "pre-clinical" or predementia or "pre-dementia" or "early stage" or "questionable Alzheimer*" or "questionable AD" or MCI or SCD or ("mild cognitive" NEAR/2 (impairment or decline or deficit or disorder)) or (subject* NEAR/2 ("cognitive decline" or "memory complaint" or "cognitive impairment")))

Supplementary Table S1.

Data collected from the selected studies

**Description of the cohort**
General
Cohort name (is applicable)
Lead author
Country in which the study was conducted
Aim of the study
Start date
End date or ongoing as of March 2023 or unknown
Inclusion criteria*
Exclusion criteria*
Participants
How are participants recruited
Sample size at baseline
Mean (SD) age at baseline
Age range at baseline*
% of women*
Education level
Follow-up time frequency and total duration*
**Clinical Diagnosis**
Diagnostic criteria for cognitively normal
Diagnostic criteria for SCD
Diagnostic criteria for MCI
Diagnostic criteria for AD
If clinical criteria change, describe
Conversion/outcome classification
Formal medical clinical evaluation (y/n)
Clinical classification disagreement handled by consensus meeting (y/n)
Diagnostic confirmation by
Use of formal neuropsychologic evaluation for diagnostic purpose*

SCD, mild cognitive impairment; MCI, mild cognitive impairment; AD, Alzheimer’s Disease
*Collected but not reported.

Supplementary Tables S2.

A. Diagnostic criteria for AD dementia at baseline per each cohort

COHORTS	DSM *	NINCDS-ADRDA	NIA-AA	CDR	ICD-10	MMSE	MOCA	Cognitive tests (other)	Clinical diagnosis
CIMAQ			■	■			■	■	
Compostela	■	■							
HELIAD	■	■							
ROSAS	■			■		■			
SHANGHAI	■	■							
BRAZIL		■							
MEMENTO									
AIBL	■	■			■				
POLAND	■								
INVECEAB	■	■							
DELCODE			■						
VIENNA		■							
GERMAN COMPETENCE		■							
KBASE	■		■	■					
SILCODE	■		■	■					
KURIHARA	■	■							
VANDERBILT									
JPSC	■	■							
BIOFINDER			■						
KLOSHA	■	■							
ADNI		■		■		■		■	
MAYO	■			■					
SYDNEY	■								
SCIENCE									■
HOPE			■						
total	14	12	6	6	1	2	1	2	1

* This includes cohorts using the DSM-III, DSM-III R, DSM IV, DSM IV-TR or DSM V editions.

B. Diagnostic criteria for MCI at baseline per each cohort

COHORTS	NIA-AA	Petersen *	Winblad-IWG	CIND	Jak & Bondi	DSM for dementia not met	CDR	MMSE	MOCA	Cognitive tests (other)	Bayer ADL	Cognitive complaints	Clinical diagnosis	No vascular burden (clinical/imaging)
ADNI		■					■	■		■				
AIBL			■							■				
BIOFINDER								■		■		■		
BRAZIL		■												
CIMAQ	■						■		■	■				
COMPOSTELA	■													
DELCODE	■													
GERMAN COMPETENCE										■	■	■		
HELIAD		■												
HOPE	■													
INVECEAB		■		■										
JPSC		■												
KBASE	■						■							
KLOSHA			■											
KURIHARA							■	■						■
MEMENTO							■			■				
POLAND		■					■							
ROSAS						■	■	■		■				
SCIENCE													■	
SHANGHAI		■												
SILCODE					■									
SYDNEY			■											
THE MAYO CLINIC STUDY		■					■							
VANDERVILT	■													
VIENNA		■												
total	6	9	3	1	1	1	8	4	1	7	1	2	1	1

* This includes cohorts using the 1997, 1999, 2001 or 2004 Petersen criteria

Supplementary Tables S2 (continued).

C. Diagnostic criteria for SCD at baseline per each cohort

COHORTS	Jessen	SMCQ questionnaire	Cognitive change index	Subjective cognitive complaints	Normal performance on cognitive tests	Criteria for MCI not met	Criteria for dementia not met	Normal cognition (clinical diagnosis)
ADNI			■	■	■			
AIBL				■				
BIOFINDER				■	■		■	
BRAZIL								
CIMAQ	■							
COMPOSTELA		■			■			
DELCODE	■							
GERMAN COMPETENCE								
HELIAD								
HOPE								
INVECEAB								
JPSC								
KBASE								
KLOSHA								
KURIHARA								
MEMENTO				■		■		
POLAND								
ROSAS								
SCIENCE	■					■	■	■
SHANGHAI								
SILCODE	■							
SYDNEY								
THE MAYO CLINIC STUDY								
VANDERVILT								
VIENNA								
total	4	1	1	4	3	2	2	1

D. Diagnostic criteria for cognitively normal individuals at baseline per each cohort

COHORTS	Absence of memory complaints	CDR	MOCA	MMSE	Normal performance on cognitive tests	Clinical criteria for MCI or dementia not met	Bayer-ADL
ADNI	■	■		■	■	■	
AIBL	■				■		
BIOFINDER	■			■		■	
BRAZIL					■		
CIMAQ	■	■	■		■		
COMPOSTELA							
DELCODE	■				■		
GERMAN COMPETENCE		■			■		
HELIAD							
HOPE							
INVECEAB							
JPSC				■	■		
KBASE		■				■	
KLOSHA							
KURIHARA		■					
MEMENTO							
POLAND							
ROSAS		■		■	■		
SCIENCE							
SHANGHAI							
SILCODE							
SYDNEY					■	■	■
THE MAYO CLINIC STUDY		■			■	■	
VANDERVILT		■			■		
VIENNA					■		
total	5	8	1	4	12	5	1
